# Deterministic recruitment limitation during soybean pod development shapes seed-transmitted microbiota assembly

**DOI:** 10.1093/ismejo/wrag183

**Published:** 2026-07-15

**Authors:** Da-Ran Kim, Min Seong Seok, Young Min Ko, Donggye Lee, Eui-Hwan Chung, Jin Hwan Lee, Kihyuck Choi, Jenog-Seon Kim, Youn-Sig Kwak

**Affiliations:** Horticultural and Herbal Crop Environment Division, National Institute of Horticultural and Herbal Science, Rural Development Administration, Wanju 55365, Republic of Korea; Division of Applied Life Science (BK21) and Research Institute of Life Science, Gyeongsang National University, Jinju 52828 Republic of Korea; Division of Applied Life Science (BK21) and Research Institute of Life Science, Gyeongsang National University, Jinju 52828 Republic of Korea; Division of Applied Life Science (BK21) and Research Institute of Life Science, Gyeongsang National University, Jinju 52828 Republic of Korea; Department of Plant Biotechnology, Korea University, Seoul 02481 Republic of Korea; Deparment of Smart Green Resource, Dong-A University, Busan 49315 Republic of Korea; Deparment of Applied Bioscience, Dong-A University, Busan 49315 Republic of Korea; Division of Agricultural Microbiology, National Institute of Agriculture Science, Rural Development Administration, Wanju 55365 Republic of Korea; Division of Applied Life Science (BK21) and Research Institute of Life Science, Gyeongsang National University, Jinju 52828 Republic of Korea

**Keywords:** ecological filtering, flower, seed microbiota, vertical transmission

## Abstract

The transgenerational inheritance of plant microbiota is fundamental to host fitness, however the ecological rules governing microbial assembly during the transition from flower to seed remain poorly understood. Therefore, we characterized the spatiotemporal dynamics of the soybean microbiota across developmental stages from vegetative to reproductive growth, and identified the specific seed-pod stages that drive a critical deterministic bottleneck for the recruitment of seed endophytes. Although the floral stage serves as a high-diversity recruitment keystone taxa dominated by stochastic processes, pod enclosure imposes rigorous host-mediated filtering and dispersal limitation, shifting the community toward a specialized “guardian” microbiota. By employing functional characterization and defined microbial community assays, we demonstrated that keystone taxa utilize niche partitioning and functional complementarity to provide collective resilience. The consortium may contribute to the reduction of salinity stress by stabilizing photosynthetic efficiency and suppresses *Phytophthora sojae* infection through synergizing direct antagonism with compensatory growth. Anatomical evidence suggests the soybean endocarp acts as a physical interface and ecological reservoir, facilitating the redistribution of maternal microbes into the maturing seed. Our findings define a precise “transmission window” for microbiota-based interventions, providing a roadmap for engineering more resilient crop–microbe associations across generations.

## Graphical Abstract

**Graphical Abstract:**
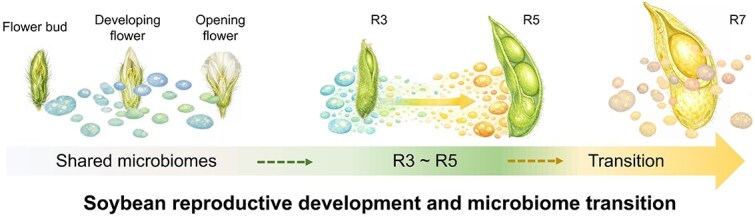

## Introduction

Ongoing climate change is intensifying land pressure and freshwater scarcity, reshaping crop production systems and threatening agricultural sustainability [1]. Global food demand is projected to rise substantially toward 2050, driven by population growth and shifts in consumption patterns [2]. Soybean is widely regarded as an important crop for future food and feed systems because of its nutritional value and broad agricultural relevance. Soybean grains provide high-quality protein and favorable lipid profiles that support human health [3]. Collectively, these attributes position soybean as a strong candidate crop in efforts toward climate-resilient and nutrition-secure agriculture [2].

Among developmental stages, flowering represents a critical point for soybean because it strongly influences pod set and final yield determination [4]. Rather than a simple physiological switch, reproductive transition involves major genetic and developmental reprogramming, including the coordinated regulation of flowering-related pathways [5]. Because climate-driven stresses increasingly threaten yield stability, understanding and ultimately managing the flowering stage and its downstream transition to pod formation still needs better understanding. Plant-associated microbiota contribute to host nutrition, hormone balance, and defense, thereby influencing growth and stress tolerance. In vegetative tissues, endophytic communities can change across growth stages, and certain taxa such as *Methylobacterium* have been linked to plant-associated traits in multiple systems [6]. Compared with the rhizosphere and vegetative organs, however, microbiota of reproductive tissues (flowers, pods, and developing seeds) remain less mechanistically resolved. This gap matters because reproductive organs experience a sharp ecological transition from an open flower environment exposed to airborne and insect-associated microbes to a more enclosed pod microenvironment. Such changes are expected to alter not only community composition but also the ecological processes driving assembly, shifting between dispersal driven stochasticity and deterministic environmental filtering [7, 8]. Consistent with this broader view, reproductive tissues across plant species can harbor a conserved subset of endophytes and may serve as reservoirs for transmission into developing seeds [9]. Floral microbiota has also attracted attention as intervention targets because flowers act as a major interface for microbial immigration and potential biological control opportunities [10].

In soybean, pod development adds another layer of constraint that may be directly relevant to microbial movement and persistence. The pod wall is structured into distinct layers, and pod traits related to dehiscence and wall properties are tightly linked to anatomical features such as fiber cap cell development and endocarp associated structures [11, 12]. More generally, flower and pod structures influence dispersal and the physical organization of developing seeds, which can shape biotic interactions within reproductive tissues [13]. These developmental and anatomical transitions create an opportunity to ask whether discrete stages act as “ecological transition points” for the reproductive microbiota and whether microbes detected early in flowers can persist or reappear later as seeds mature [9].

Synthetic microbial community provides an experimental framework to test causal roles of microbiota members by assembling simplified communities from plant-associated strains and applying them under controlled conditions. In this study, the term “Defined Microbial Community (DMC)” is adopted in place of “Synthetic Community (SynCom)”, as the term “synthetic” carries potential negative connotations associated with genetic engineering and synthetic biology, and may contribute to public skepticism toward microbiota-based applications [14]. Recent work emphasizes designing DMC in ways that preserve ecological structure and improve reproducibility of host phenotypes, rather than simply combining individually beneficial strains [15]. These experiments often employ gnotobiotic or highly standardized systems to reduce environmental noise and enable robust inference of microbial effects on host traits [16]. Such approaches are particularly useful when functional effects may depend on interactions among strains and on the developmental context of the host [15, 16].

Here, we investigated stage-dependent dynamics of the soybean reproductive microbiota from flowering to seed maturation and test how microbiota informed DMC formulations affect soybean performance under abiotic and biotic stresses. Specifically, we profiled microbial community succession across flower to seed developmental transitions and quantify shifts in ecological assembly processes using phylogenetic and dispersal based null modeling [7]; characterize functional traits and antagonistic activities of representative DMC strains; and evaluate how DMC composition modulates soybean responses to stress, linking reproductive microbiota assembly to experimentally testable microbial functions and plant phenotypes [15, 16].

## Materials and methods

### Experimental design and sampling strategies for small-scale and large-scale analyses

Soybean (*Glycine max,* cv. Daepung) seeds were surface sterilized by placing 10 g of seeds in a 50 ml conical tube filled with 1% (v/v) sodium hypochlorite (NaClO) solution and reacting for 10 min. After removing the NaClO solution, the seeds were rinsed five times with sterile distilled water (ddH_2_O). The surface-sterilized seeds were transferred to a Petri dish lined with sterile cotton and cultured in the dark at 28°C for 3 days for germination. The germinated seedlings were transplanted into 9 × 9 cm plastic pots filled with horticultural growing medium, with one seeding established per pot. The plants were grown in a greenhouse with a photoperiod of 14–16 h at 28°C ± 3°C and a dark cycle of 8–10 h at 15°C ± 3°C, uniform tap water irrigation. A total of 60 plants were used in this study, consisting of 30 seedlings per replicate across two biological replicates. Samples were collected across specific developmental stages defined by flower and pod development stages.

To investigate microbiota dynamics across soybean reproductive development, sampling was conducted using a two-tier experimental design consisting of a small-scale exploratory analysis followed by a large-scale validation analysis. For the small-scale analysis, soybean flower, pod, seed, and stem tissues were collected to capture fine-scale developmental transitions. Flower were harvested during the flowering stage, encompassing flower bud (FB) and beginning flower (BF) stages, followed by developing flower (DF) and opening flower (OF) stages. Pod-stage samples corresponding to R2 (pollinated flower/initiating pod) were collected. Seed tissues were collected across the R4 (developing seed) and R5 (mature seed) stages. In addition, stem endosphere samples were collected from young stems representing vegetative xylem (VX), and from old stems representing reproductive xylem (RX). Samples for the small-scale analysis were collected from five independent plants, resulting in five biological replicates for each developmental stage and tissue type.

Based on the patterns of results identified in the small-scale dataset, four representative stages (flower bud, flower, R3, and R5) were selected for large-scale analyses. Large-scale sampling was conducted according to the same dates and developmental criteria as the small-scale experiment to ensure seasonal and developmental consistency. Large-scale samples were collected from 30 independent plants for the four representative stages, with 30 biological replicates per stage. Tissue collection, DNA extraction protocols, and subsequent microbiota community analyses were identical to those in the small-scale experiment. The small-scale analysis allowed for high-resolution identification of microbial transition points during soybean reproductive development, allowing for statistical validation of key stage-specific clustering patterns in the large-scale dataset (Table S1).

### DNA extraction and microbial community analysis

Flower stage, pod stage, and stem tissues were sampled for microbiota analyses. For flower tissues (flower bud, beginning flower, developing flower, and opening flower), ~0.2 g of tissue per sample, was used for DNA extraction. For pod stages, 0.2 g per sample was used at the R2 stage, 5–6 pods at the R3 stage, and 2–3 pods at the R4 and R5 stages, respectively. For stem endosphere samples, 0.3 g of tissue per sample was collected from young stems (VX) and old stems (RX), corresponding to 11–15 stems per sample. All samples were surface-sterilized by sequential immersion in 1% NaClO for 30 s, followed by 70% ethanol for an additional 30 s, and rinsed three times with sterile distilled water (ddH_2_O) to remove surface contaminants. Sterilized tissues were then submerged in 1× phosphate-buffered saline (PBS; 8 g NaCl, 2 g KCl, 0.144 g Na_2_HPO_4_, 0.24 g KH_2_PO_4_ per L, pH 7.4) and sonicated for 15 min at 35 kHz to dislodge remaining surface-associated microbes [17]. After sonication, tissues were again treated with 70% ethanol and 1% NaClO for 30 s each, followed by two rinses in sterile ddH_2_O. Flower tissues were air-dried at room temperature for 15 min, whereas pod and stem tissues were air-dried for 1 h in a sterile laminar flow hood [18].

Microbial DNA was extracted from the prepared tissues using the FastDNA SPIN Kit (MP Biomedicals, Irvine, CA, USA) according to the manufacturer’s instructions. Extracted DNA concentrations were normalized to 5–8 ng/μL prior to downstream analyses. The V3–V4 regions of the bacterial 16S rRNA gene were amplified using primers 515F (5′-GTGCCAGCMGCCGCGGTAA-3′) and 805R (5′-GACTACHVGGGTATCTAATCC-3′) containing Illumina adapter overhangs [19]. Peptide nucleic acid (PNA) blockers targeting plant chloroplast and mitochondrial DNA (pPNA and mPNA) were added to all endosphere samples, whereas rhizosphere samples were amplified without PNA blockers [20]. PCR reactions (40 μL total volume) contained 2.5 μL of template DNA, 1 μL of each 16S rRNA primer (10 pmol), 2.5 μL of each PNA probe (7.5 μM, endosphere only), 12.5 μL of KAPA HiFi HotStart ReadyMix (Roche, Basel, Switzerland), and nuclease-free water. The thermal cycling conditions consisted of an initial denaturation at 95°C for 3 min, followed by 25 cycles of denaturation at 95°C for 30 s, PNA annealing at 78°C for 10 s (endosphere only), primer annealing at 55°C for 30 s, and extension at 72°C for 30 s, with a final extension at 72°C for 5 min. PCR amplicons were purified using AMPure XP beads (Beckman Coulter, Brea, CA, USA), quality-checked via 1% agarose gel electrophoresis (100 V, 20 min), and sequenced using 2 × 300 bp paired-end reads on the Illumina MiSeq platform at Macrogen Inc. (Seoul, Korea).

Raw sequencing data were processed using the DADA2 pipeline (v1.20.0) in R (v4.1.3), which included quality filtering, error correction, merging of paired-end reads, and chimera removal to generate amplicon sequence variants (ASVs) [21]. Taxonomic assignments were performed using the SILVA database (v138) and the IDTAXA classifier with a 99% identity threshold [22, 23]. Alpha diversity indices (ACE, Fisher, InvSipmson, Observed ASVs, Shannon, Simpson) and beta diversity based on Bray–Curtis dissimilarity were computed using the vegan (v2.6–2) and phyloseq (v1.36.0) R packages. Community richness and evenness were assessed using ggiNEXT (v2.0.20) and phyloseq [24]. Principal Coordinates Analysis (PCoA) was applied to visualize dissimilarities in microbial communities based on Bray–Curtis metrics. All plots were generated using ggplot2 (v3.3.5) [25]. Statistical significance in diversity metrics was determined using Kruskal–Wallis tests with a threshold of *P* < .05.

Weighted βNTI and βRC matrices were generated for pairwise comparisons among samples across different developmental stages and plant compartments (flower bud, immature/mature flower, opening flower, R2–R5 pod stages, young/old stem) [26]. Taxa with unidentified or undefined values were grouped into NA. The βNTI and βRC approaches used stacked bar charts to visualize the relative importance and stage-specific patterns of microbial community dynamics during soybean development. Ternary plots were constructed using the ggtern (v4.5) package in R to visualize three-group comparisons. ASVs were taxonomically classified against the SILVA SSU database (v138, 2019) using the IDTAXA classifier from the DECIPHER package, and clustered into ASVs. Relative abundance values were calculated by normalizing ASVs counts within each group, and average abundance across comparisons was used to scale marker sizes. Visualization was performed using phylum-level classification, with color representing each ASV, and marker size representing the average relative abundance of the three groups. Low-abundance taxa (<0.5%) were identified as the “Other” group. The sources used in the analysis were the SourceTracker package [27], following the implementation described in the original repository (https://github.com/danknights/sourcetracker/). ASV-based taxonomic tables were generated, in which flower bud samples were assigned into sources, although flower and pod samples at the R3 and R5 stages were assigned within sinks.

### Bacterial strains and growth condition

Bacterial strains utilized in the DMC included *Variovorax paradoxus* NBRC15149 (Var) and *Rhodococcus* sp. KCB6 (Roc), *Streptomyces globisporus* TFH56 (TFH), and *Pseudonocardia autotrophica* DMS535 (Pse). Roc was cultured in R2A agar media (R2A broth 3.15 g, agar 20 g per L), TFH and Pse were propagated in MS agar media (mannitol 20 g, soya flour 20 g per L) applying a three-phase streaking technique for collection of single colonies. A sterile loop was used to inoculate Var and Roc into 5 ml of liquid R2A medium, and then pre-cultured for two days at 28°C, and at 250 rpm. The pre-culture was transferred to 500 ml of liquid R2A medium and cultured under same conditions. Bacterial cells were harvested at 4°C using a centrifuge provided with a GRS L-r250–4 rotor (radius 17.52 cm, rectangular bucket; Hanil Science Industrial, Korea) at 3134 × g for 40 min. After centrifugation, the supernatant was discarded, and the cell pellet collected was washed twice using sterile ddH_2_O in similar conditions to fully eliminate the excess medium components.

Single colonies of TFH and Pse were obtained and streaked on the surface of MS medium and incubated for 7 days to generate spores. Harvesting spores was performed by placing two sterile absorbent cotton (1 cm × 1 cm) on the medium plate, and 2 ml of sterile distilled water was gradually added to fully absorb the spores. The spore-absorbed cotton pads were transferred to a new sterile 10 ml sterile syringe with a clean cotton pad pre-inserted into it. which was then all compressed and squeezed carefully to transfer a fresh spore stock solution to a new 50 ml conical tube. The concentration of the prepared spore suspension was adjusted to 10^7^ cfu/ml for Var and Roc, and 10^6^ cfu/ml for TFH and Pse.

### Functional characterization of defined microbial community strains for plant growth promotion and pathogen antagonism

DMC strains selected during the flowering and pod development stages of soybeans are highly potential to contribute to plant health. The potential for plant-microbe symbiosis and antagonistic activity against pathogens was assessed by conducting six enzyme activity assays.

First, proteinase activity was assessed to confirm the antagonistic activity of the combined DMC strains against pathogens. Skim milk agar medium (skim milk powder 10 g, agar 20 g per L) was prepared and each strain was calibrated to OD_600_ = 0.3 [28]. A sterilized filter disc was placed at the center of the medium surface, inoculated with 10 μL of the bacteria cell suspension, and cultured at 28°C for 2 days. Protease activity was then assessed by measuring the diameter of the formed clean zone with the ImageJ software (version 1.54) [29].

The phosphate solubilization assay was performed to determine the potential for symbiosis with plant roots. Pikovskaya medium (KCl 0.2 g, MgSO_4_-7H_2_O 0.1 g, glucose10 g, yeast extract 0.5 g, of FeSO_4_-7H_2_O 0.002 g, MnSO_4_ 0.002 g, (NH_4_)_2_SO_4_ 0.5 g, and Ca_3_(PO_4_)_2_ 5 g per L) was prepared [30]. Strains were adjusted to OD_600_ = 0.3 and inoculated 10 μL onto a filter disk in the center of the medium, and the clean zone formed after 14 days of incubation was identified and its diameter measured with the ImageJ software.

Evaluation of siderophore production capacity was performed using Chrome Azurol S (CAS) based medium to determine the ability to compete with pathogens for Fe nutrition [31]. Casamino acids solution (casamino acids 3 g/30 ml) and 3% 8-hydroxyquinoline/chloroform solution (0.3 g/10 ml) were prepared, mixed 1:1 and reacted overnight at 4°C. The medium was adjusted to pH 6.5 by mixing 100 ml of MM9 salt solution (KH_2_PO_4_ 30 g, NaCl 50 g, NH^4^Cl 100 g per L), 750 ml of DW, 32.24 g of PIPES, and 20 g of agar, and then sterilized. When the medium had cooled to 50°C, 30 ml of CAS supernatant, 10 ml of 20% glucose stock, and blue dye solution (chrome azurol S 0.6 g, FeCl_3_-6H_2_O 0.027 g, hexadecyltrimethylammonium bromide 0.73 g per L) were added [32]. A filter disk was placed in the center of the medium and inoculated with 10 μL of the strain suspension calibrated to OD_600_ = 0.3, and the clean zone formed after 7 days was observed.

The nitrogen-fixing capacity assessment was performed on nitrogen-free bromothymol blue semi-solid medium (malic acid 5 g, K_2_HPO_4_ 0.6 g, KH_2_PO_4_ 0.4 g, NaCl 0.02 g, of Na_2_MoO_4_-2H_2_O 0.002 g, MnSO_4_-H_2_O 0.01 g, 0.5% bromothymol blue in 2 ml ethanol, 1.75 g agar per L, pH 6.6–7.0) was prepared and aliquoted and sterilized to 5 ml per test tube. The strain was calibrated to OD_600_ = 0.3 and inoculated into the upper two-thirds of the medium using a loop. After 7 days of incubation, the strain was checked for a color change from green to blue and absorbance was measured at OD_600_ compared to the control [33].

The assessment of indole-3-acetic acid (IAA) production was performed using the Salkowski reagent. L-tryptophan solution (1%) was prepared, filtered through a 0.2 μm filter, and adjusted to a final 0.1% by adding 1% L-tryptophan to R2A medium [34]. The prepared medium was dispensed in 20 ml aliquots into 50 ml tubes and incubated for 5 days, although the control was incubated in R2A medium without L-tryptophan. The cultures were centrifuged at 3393 × g, 40 min to recover the supernatant, and the supernatant was mixed with Salkowski’s reagent (2% of 0.5 M FeCl3 in 35% HClO_4_ solution) in a 1:2 ratio. The mixture was reacted for 30 min at 28°C, 250 rpm, and the pink color development was measured at OD_530_.

Evaluation of chitinase activity was performed in chitinolytic reaction medium (colloidal chitin 5 g, yeast extract 5 g, of KH_2_PO_4_ 0.7 g, of K_2_HPO_4_ 0.05 g, of MgSO_4_ 0.3 g, of FeSO_4_ 0.1 g, NaCl 0.1 g and agar 20 g per L, pH 6.5–7.0) was used [35]. A filter disk was attached to the center of the medium and 10 μL of strain suspension adjusted to OD_600_ = 0.3 was dispensed. After 7 days of incubation at 28°C, 1 ml of 0.2% Congo red was added, stained and dried for 30 min, and washed with 1 ml of 1 M NaCl in three aliquots. The formed clean zone was measured with ImageJ software.

### Preparation of defined microbial community-coated soybean seeds for germination assays

Thirty dried seeds were transferred to sterile tubes for each treatment, weighing ~10 g. DMC bacterial stocks (Var and Roc: 1 × 10^7^ CFU/ml; TFH and Pse: 1 × 10^6^ CFU/ml) were mixed with 0.1% carboxymethyl cellulose (CMC; Sigma-Aldrich, USA) solution. The bacterial stock and CMC mixture used for seed coating was applied at a ratio of 10 μL per 1 g of seed weight. The bacterial stock and CMC mixture was applied to a sterile tube containing dried soybeans and shaken vigorously to prevent liquid from adhering to the walls. The coated seeds were spread on a 9 cm sterile petri dish and air-dried at room temperature for 1 h. Sterile cotton was placed on the sterile petri dish, 20 ml of sterile distilled water was added, and the treated seeds were placed on top. To promote germination under dark conditions, seeds were sealed with aluminum foil and stored in an incubator at 27°C for 3 days. The following seed coating method was applied equally to all single, double, and multiple strain DMC treatments in subsequent salt stress and growth assays.

To evaluate the growth-promoting efficacy of DMC compositions, soybean seedlings germinated after seed coating were transplanted into 9 cm pots containing medium. Experimental groups included single-strain, two-strain, multi-strain DMC treatments, with 0.1% CMC used a control. Seedlings were cultured under conditions of 30°C/28°C (day/night), a 16-h photoperiod (16 h light/8 h dark), and 50% or higher relative humidity. Seven days after transplanting, plants were harvested, and the length and weight of the shoot and root were measured. The point where the first root branched was used to define the boundary between the shoot and root. For the above-ground weight measurement, all leaves and lateral branches except the main stem were removed, and only the main stem weight was measured. To test the statistical significance of the measured data, Shapiro–Wilk and Bartlett tests were used to confirm normality and homoscedasticity, respectively. As the analysis results did not meet the parametric statistical assumptions, the nonparametric Kruskal–Wallis test was performed, and a *post hoc* test was performed using Conover’s *post hoc* test. Statistical significance was indicated by letters.

### Evaluation of defined microbial community-mediated abiotic stress tolerance in soybean

DMC strains (Var and Roc: 1 × 10^7^ CFU/ml; TFH and Pse: 1 × 10^6^ CFU/ml) were coated onto seeds in single, double, triple, and all mixed sets of treatment (Table S2). Soybean seedlings vernalized for 3 days were transferred to 9 cm pots and transplanted into soil. To provide necessary nutrients during the plant growth period, diluted (1/10) Hoagland solution (6.0 mM KNO_3_, 4.0 mM Ca(NO_3_)_2_·4H_2_O, 1.0 mM NH_4_H_2_PO_4_, 2.0 mM MgSO_4_·7H_2_O, 46.3 μM H_3_BO_3_, 9.1 μM MnCl_2_·4H_2_O, 0.32 μM CuSO_4_·5H_2_O, 0.77 μM ZnSO_4_·7H_2_O, 0.12 μM Na_2_MoO_4_·2H_2_O, 100 μM Fe(III)-EDTA per L) was watered [36].

Plants were grown within 16-h photoperiod (16 h light/8 h dark) at room temperature for 2 weeks. 160 mM NaCl was added to 1/10 Hoagland solution to elicit salt stress, and 1.5 L of the solution was administered to each treatment group (*n* = 7) categorized in separate trays. After 4-day treatment, analysis of salt stress injury (SI) index was conducted based on the specific time point when the preliminary wilting symptoms were detected in the control. Damage assessment was divided into five levels (level 0–4) of salinity injury index in accordance with the criteria described in a previous study [37]. Level 0: absence of visible stress symptoms; Level 1: presence of yellow discoloration in leaves, and delayed development; Level 2: onset of drying of leaf tips and wilting of plants; Level 3: completely dried and stems began drying; Level 4: complete drying and wilting of plants. SI value was calculated using the following formula:

The salt injury (SI) index = (N1 + N2 ×2 + N3 ×3 + N4 ×4)/(N ×4) ×100. Additionally, quantification of the electron transport flux per reaction center (ETo/RC) and the dissipated energy flux per reaction center (DIo/RC) in leaves situated directly below the uppermost node of the stem in five plants per treatment group were analyzed utilizing the photosynthetic fluorescence meter FluorPen FP110 (Photon Systems Instruments, Czech Republic). The acquired data were subjected to Shapiro–Wilk normality tests and Bartlett’s tests for homogeneity of variance. If the assumptions were not satisfied, nonparametric tests (Kruskal–Wallis analysis) and Conover *post hoc* tests were applied to evaluate statistical significance.

### Biocontrol assay of defined microbial community strains against *Phytophthora sojae*

*Phytophthora sojae* (KACC no. 48989), the major soybean pathogen, was utilized to verify biotic response of DMC strains. Preparation of V8 agar media (CaCO_3_ 2 g, V8 juice 200 ml, agar 20 g, distilled water 800 ml per L) was made followed by bacterial incubation at 27°C for 7 days to attain complete growth of pathogens on the plate media. Among various combination of DMC strains, Var and Pse (OD_600_ of 0.5), and TFH and Roc (OD_600_ of 0.3) were chosen in subsequent analyses. All strains were diluted 1:10 in a 0.1% CMC solution (10 g of CMC per L) solution and used in experimental assays. Nine cm pots were filled with potting soil followed by sowing of soybean seeds. Eight treatment groups each containing 10 plants were prepared: four single-strain treatments, a multi-strain treatment (combination of all four strains), a 0.1% CMC treatment (control), a distilled water (DW) treatment (control), and a pathogen-free treatment (control) (Table S3). Pathogen inoculation was applied in all treatment excluding the pathogen-free treatment group. The specific strain suspension (15 ml) or control solution was administered into each treatment group on the day of sowing, and the treatment was repeated three times at 2-day intervals. No water was added to the plants during the treatment period.

Inoculation of *P. sojae* was conducted at six days post-sowing of plants upon emergence of the true leaves. Using a sterile scalpel, bacterial strains grown in V8 medium was finely chopped then squeezed twice through a 50 ml syringe equipped with an 18G needle to remove the excess agar. Subsequently, application of 200 μL suspension into each plant was performed using a 10 ml syringe equipped with an 18G needle. Using a syringe needle, a laceration was made located at the boundary between the aboveground and underground parts of the soybean, followed by the injection of the medium [38].

Assessment of the disease symptoms was performed considering the following seven levels: Level 1, no symptoms (healthy plants); Level 2, lesion length < 1 cm; Level 3, lesion length ≥ 1 cm; Level 4, stems wilted and leaves yellowed, but no lodging observed; Level 5, leaves yellowed with lodging; Level 6, leaves completely dried with lodging; Level 7, plants completely dead [38]. Root measurement was quantified using ImageJ software (version 1.54) after plant photographs were taken. Disease index and root length measurements undergo statistical analysis using the Kruskal–Wallis test and Conover *post hoc* test after verification of the normality and homogeneity of variance utilizing the Shapiro–Wilk and Bartlett tests.

## Results

### Microbiota assembly dynamics across flower to seed developmental transitions

**Figure 1:**
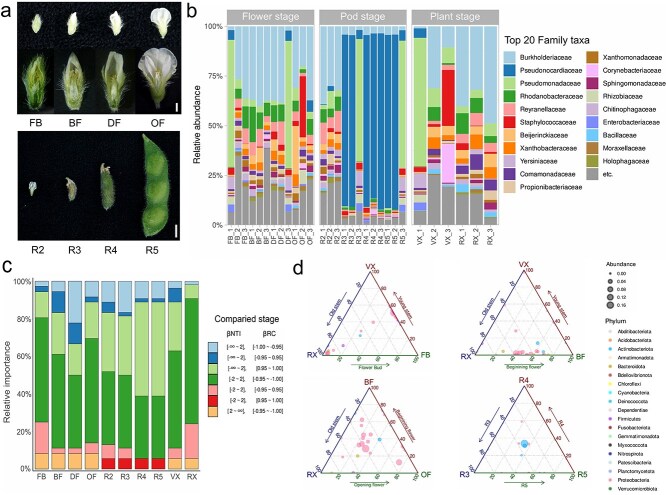
Reproductive-stage progression in soybean is accompanied by a transition from shared to specialized microbiota. (a) Representative images of soybean reproductive sampled across flower and pod developmental stages, including flower bud (FB), beginning flower (BF), developing flower (DF), opening flower (OF), and pod stages R2–R5. Young stem (VX) and old stem (RX) were additionally sampled to stem endosphere variation. (b) Relative abundance of dominant bacterial families across developmental stages. ASVs were generated using the DADA2 pipeline (v1.20.0) in R (v4.1.3), taxonomically assigned against the SILVA SSU database (v138) using the IDTAXA classifier, and normalized prior to visualization. (c) Community assembly processes inferred using a weighted β-nearest taxon index (βNTI) combined with Bray–Curtis–based Raup–Crick (βRC) analysis. Pairwise sample comparisons were classified into homogeneous selection, heterogeneous selection, dispersal limitation, homogenizing dispersal, or stochastic processes based on established βNTI and βRC thresholds, and summarized as stacked bar plots. (d) Ternary plots depicting the distribution of ASVs across selected developmental stage comparisons. Ternary visualizations were generated using the ggtern package (v4.5) in R, with marker size scaled to mean relative abundance and color indicating phylum-level taxonomy. ASVs with relative abundance below 0.5% were excluded from visualization. Bray–Curtis dissimilarity matrices were calculated using the vegan package (v2.6–2) and phyloseq (v1.36.0). Community richness and evenness were assessed using ggiNEXT (v2.0.20). PCoA was performed based on Bray–Curtis distances, and all plots were generated using ggplot2 (v3.3.5). Samples represent five biological replicates per stage from the small-scale analysis**.**

Microbial community succession across soybean reproductive development was examined using a two-step approach consisting of an initial small-scale screen followed by large-scale validation. In the small-scale analysis, microbiota communities were profiled across eight developmental stages from flower (FB, BF, DF, OF) to pod (R2, R3, R4, R5), as well as young (VX) and old (RX) stem tissues, with five biological replicates per stage (Fig. 1a).

Rarefaction curves generated from ASV tables showed that observed richness increased with sequencing depth and generally approached saturation across sample groups, supporting that the sequencing effort captured a substantial fraction of microbiota diversity across reproductive stages and stem endosphere tissues (Fig. S1a). Consistent with these quality and diversity assessments, multiple alpha diversity indices (ACE, Fisher, InvSimpson, Observed ASVs, Shannon, and Simpson) were calculated across developmental stages and tissue types, enabling stage wise comparisons of richness and evenness in flower, pod, and stem-associated communities (Fig. S1b and c). Group level differences in these diversity metrics were statistically evaluated using Kruskal Wallis tests (*P* < .05 threshold), providing complementary support for stage associated structuring of microbial communities during reproductive development (Fig. S1b and c). This initial screen revealed pronounced stage associated shifts in microbial community composition. At the family level, flower associated communities were dominated by Burkholderiaceae, Pseudomonadaceae, and Sphingomonadaceae, whereas pod associated communities exhibited a distinct enrichment of Xanthomonadaceae and Comamonadaceae, particularly at R3 and R5 (Fig. 1b). Among pod stages, R3 showed the lowest within stage variability across replicates, suggesting a relatively constrained community structure during early pod development (Fig. 1b).

Analysis of community assembly mechanisms using βNTI and βRC further indicated that the relative contribution of stochastic and deterministic processes differed across developmental stages. Flower stage communities were primarily shaped by stochastic processes, supported by indicated by βNTI values between −2 and 2 and βRC values close to zero (Fig. 1c). These patterns are consistent with high dispersal and random drift in an open flower environment. In contrast, R3 communities showed strong signals of homogeneous selection (βNTI < −2), accounting for ~60%–70% of inferred assembly processes (Fig. 1c). This shift suggests that early pod development imposes stronger environmental filtering, likely associated with pod closure and the formation of a more restricted internal microenvironment. At R5 stage, the influence of selection was reduced, and community assembly was more strongly associated with dispersal related processes, resembling patterns observed in RX (Fig. 1c). The transition indicates partial relaxation of selection during seed maturation and redistribution of microbial taxa toward maternal vascular-associated communities.

Consistent with βNTI/βRC results, ternary plot analyses revealed dynamic redistribution of ASVs across reproductive stages. Multiple ASVs affiliated with Actinobacteriota and Proteobacteria were shared between flower-associated samples and R5, but were rare or absent at R3 stage (Fig. 1d). This pattern presents a transient exclusion of shared taxa during early pod development, followed by partial re-establishment of a seed associated microbiota overlapping with flower derived communities. To visualize fine scale community variation across the full set of small-scale stages, Bray–Curtis distance matrices were further summarized by PCoA. This ordination framework enabled comparison of community composition among flower, pod, and stem tissues across developmental progression, revealing clear clustering patterns consistent with stage- and tissue-associated shifts observed in taxonomic profiles (Fig. S2a). The regression-based modeling was applied to examine the relationship between microbial community variation and developmental stage, and model comparisons were evaluated via ANOVA to test whether explanatory structures differed across groups (Fig. S2b). Together, these analyses reinforce that reproductive progression is accompanied by coordinated shifts in β-diversity that are not limited to pairwise stage contrasts but extend across the full developmental series.

Finally, ternary plot visualizations were expanded to depict the relative distribution of microbial taxa among multiple three-way stage/tissue comparisons, with marker sizes scaled to mean relative abundance and phylum level classification used for color assignment. This analysis consistently highlighted dynamic redistribution of taxa across reproductive development although excluding/aggregating low abundance taxa (<0.1%), supporting the conclusion that community membership is transiently reweighted during early pod development and reorganized again during later maturation (Fig. S2c–h).

### Large-scale validation of microbiota convergence during seed maturation

**Figure 2:**
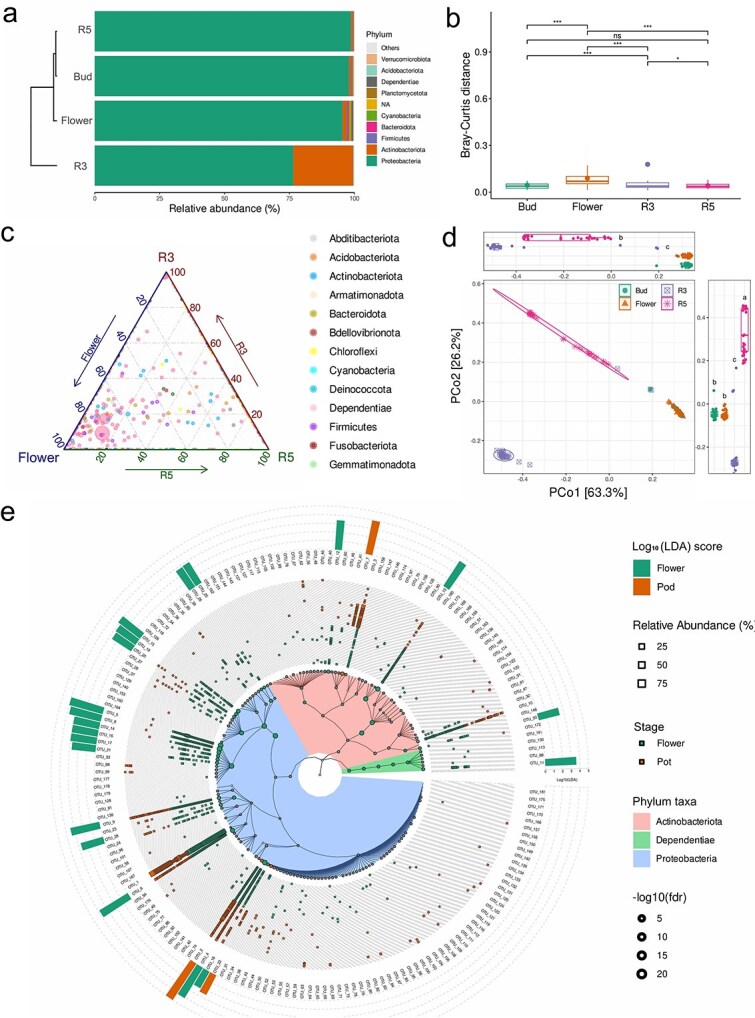
Microbiota composition shifts sharply at early pod formation across reproductive development. Bacterial community composition was analyzed across four representative developmental stages—flower bud (FB), flower, R3, and R5—using 30 biological replicates per stage. (a) Relative abundance of dominant bacterial phyla across stages, calculated from ASVs tables generated using the DADA2 pipeline (v1.20.0) and taxonomically assigned with the SILVA database (v138). (b) Bray–Curtis dissimilarity within and between stages, computed using the vegan package (v2.6–2). Statistical significance was assessed using the Kruskal–Wallis test followed by Conover’s *post hoc* comparisons; significance levels are indicated (*P* < .05, *P* < .01, *P* < .001). (c) Ternary plot showing the distribution of shared and stage-enriched ASVs among flower, R3, and R5 samples. Marker size reflects mean relative abundance, and colors denote phylum-level classification. Ternary plots were generated using the ggtern package (v4.5). (d) PCoA based on Bray–Curtis dissimilarity, visualized using phyloseq (v1.36.0) and ggplot2 (v3.3.5). The percentage of variation explained by each axis is shown. (e) LEfSe cladogram highlighting phylogenetically structured taxa discriminating between flower-associated and pod-associated microbiota. Linear discriminant analysis (LDA) scores indicate taxa with significant stage specificity, with bar colors denoting enrichment in flower or pod stages and node colors representing phylum-level affiliation.

Based on the small-scale screening results, four representative stages (Bud, Flower, R3, and R5) were selected for large-scale validation with 30 biological replicates per stage. Across all stages, Proteobacteria and Actinobacteriota dominated the microbial communities, with a marked enrichment of Proteobacteria at R3 and a partial convergence of R5 communities toward a flower bud like composition (Fig. 2a). Bray–Curtis dissimilarity analyses showed significantly greater distances between flower associated samples and R3 than between flower and R5, indicating divergence at early pod formation followed by partial convergence during seed maturation (Fig. 2b). Ternary plot analyses of the large-scale dataset corroborated the small-scale findings, revealing that multiple ASVs affiliated with Actinobacteriota and Proteobacteria were shared between flower associated samples and R5, but were depleted at R3 (Fig. 2c; Fig. S3). PCoA further supported this finding, with R3 samples forming a distinct cluster relative to flower associated samples and R5 (PCoA1 = 63.3%, PCoA2 = 26.2%; Fig. 2d). Differential abundance analysis using LEfSe identified several pod enriched taxa, including members of Rhodanobacteraceae and Comamonadaceae, which were significantly associated with early pod stages (Fig. 2e; Tables S4 and S5).

**Figure 3:**
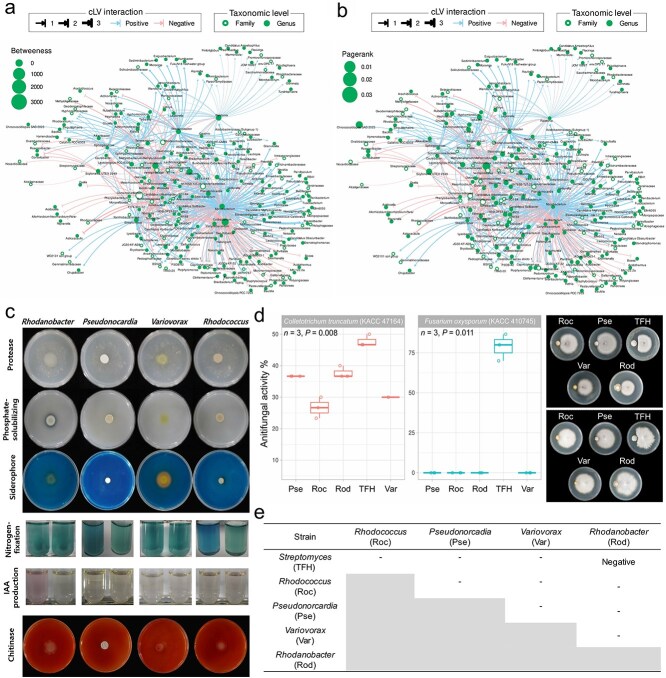
Functional traits and antagonistic capacities of DMC strains. (a and b) Microbial co-occurrence networks constructed from large-scale soybean microbiota datasets. Networks were generated based on pairwise correlations among ASVs and visualized using centrality metrics. Node size represents betweenness centrality (a) or PageRank score (b), reflecting the relative importance of each sequences ASVs within the network structure. Nodes are colored according to taxonomic affiliation, and edges indicate significant positive or negative associations. ASVs exhibiting high betweenness or PageRank values were defined as key or hub taxa and selected as candidate members for DMC construction. (c) Functional traits of DMC strains evaluated using plate- and liquid-based assays. Protease activity was assessed on skim milk agar by measuring clear zone diameters after 2 days of incubation at 28°C. Phosphate solubilization was evaluated on Pikovskaya agar following 14 days of incubation. Siderophore production was detected using chrome azurol S (CAS) agar based on halo formation after 7 days. Nitrogen fixation potential was assessed on nitrogen-free bromothymol blue semi-solid medium based on color change and absorbance relative to controls. IAA production was quantified using the Salkowski reagent after 5 days of growth in R2A medium supplemented with L-tryptophan. Chitinase activity was evaluated on chitin-containing agar stained with Congo red, and clear zones were measured after 7 days. All assays were performed using strain suspensions adjusted to OD_600_ = 0.3. (d) Antagonistic activity of individual DMC strains against fungal pathogens. Inhibition assays were conducted against *Colletotrichum truncatum* (KACC 47164) and *Fusarium oxysporum* (KACC 410745), and antifungal activity was calculated as the percentage reduction in mycelial growth compared with untreated (*n* = 3). Statistical differences among treatments were assessed using Kruskal–Wallis tests followed by Conover’s *post hoc* comparisons antagonistic capacities across DMC strains. (e) The matrix illustrates the feasibility of co-culturing two microorganisms and the relative strength of their interactions.

**Figure 4:**
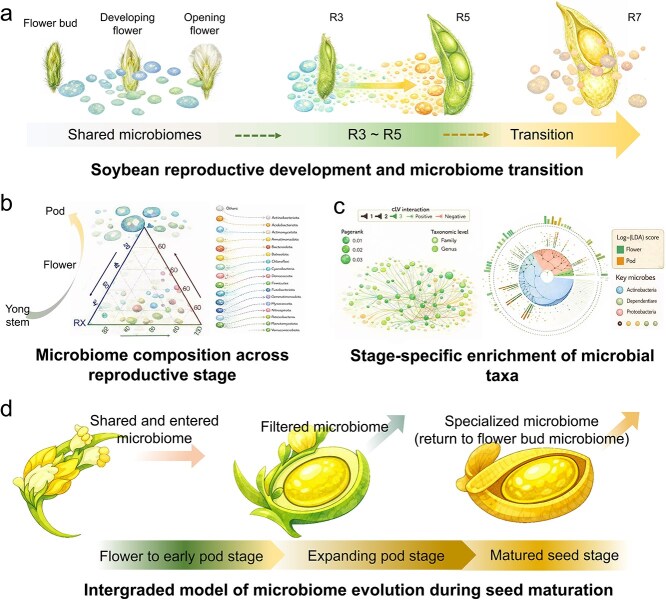
Conceptual model of inferred microbial transfer from pod to seed during soybean development. (a–d) The schematic was constructed using Affinity Designer (version 1.10) by combining vector-based graphical elements with representative soybean developmental images. All visual components were assembled to reflect analytical results rather than direct observation of microbial movement, and the illustration serves as a conceptual summary of the study framework.

To further infer potential microbial sources across reproductive development, SourceTracker analysis was applied using flower bud samples as sources and flower, R3, and R5 samples as sinks. This analysis indicated that early pod (R3) communities were largely decoupled from flower-derived sources, whereas R5 communities showed increased contributions from flower associated microbiota (Fig. S4). These results support a model in which the developing pod interior serves a selective bottleneck during early pod formation, followed by partial realignment with maternal microbial reservoirs during later maturation (Fig. 3a; Fig. S4). An integrative schematic summarizes the proposed microbiota transmission and filtering framework across flower to seed development (Fig. 4). Briefly, microbes colonizing the flower surface can enter the endoscarp/pod interior during fertilization and early pod formation, where environmental filtering is strongest (R3). During later maturation, redistribution and/or recolonization from maternal tissues (including xylem associated communities) may contribute to the partial convergence observed at R5 (Fig. 4).

### Functional traits and antagonistic activities of keystone strains

To functionally characterize the keystone taxa members identified from microbiota network analyses (Fig. 3a and b; Tables S6 and S7), The representative strains were evaluated for enzymatic activities and plant growth–associated traits. The strains exhibited distinct and largely non-overlapping functional profiles, indicating functional complementarity rather than redundancy (Fig. 3c and e; Tables S8 and S9). *Streptomyces globisporus* displayed chitinase activity and moderate protease activity, whereas phosphate solubilization and nitrogen fixation were not detected. *Rhodococcus* sp. lacked detectable chitinase and protease activity but exhibited nitrogen fixation potential (Fig. 3c and e; Table S10). *Rhodanobacter spathiphylli* exhibited pronounced phosphate-solubilizing activity and IAA production, although chitinase and protease were not detected. Siderophore production varied among strains and was most prominent in *Variovorax* and *Pseudonocardia* (Fig. 3c and e; Table S10).

Antagonistic activity against fungal pathogens also differed among strains (Fig. 3d). Against *Colletotrichum truncatum*, a causal agent of soybean anthracnose disease, inhibition rates varied significantly (*P* = .008), with *Streptomyces* and *Rhodococcus* showing stronger antifungal activity than *Pseudonocardia* and *Variovorax.* In assays against *Fusarium oxysporum,* a pathogen of soybean wilt disease, only *Streptomyces* exhibited measurable inhibition (*P* = .011). Collectively, these results demonstrate that individual keystone strains contribute distinct functional traits related to nutrient acquisition, hormone production, and pathogen suppression, supporting the rationale for multi-member DMC assembly. Inter-species competition assays revealed that *Streptomyces* and *Rhodanobacter* inhibited each other’s growth. Therefore, a final DMC was constructed excluding *Rhodanobacter*, consisting of four strains: THF, Roc, Pse, and Var (Fig. 3e).

### Defined microbial community composition modulates early seedling establishment and growth promotion ability

To elucidate the effects of DMC strains on early soybean seedling establishment and growth, we analyzed the indices of shoot and root growth of seedlings germinated after seed coating (Fig. S5a and b). The untreated control group was treated with 0.1% CMC. Seven days after transplanting, the length and weight of each part were measured based on the point of first root branching. Analysis of seedling growth responses in single-strain treatments revealed no statistically significant differences in shoot length and root length compared to the untreated group (Fig. S5c and e). Fresh weight indices showed differential patterns among strains. TFH tended to decrease both shoot and root weights compared to the untreated group, suggesting that single treatment may have a somewhat inhibitory effect on early growth (Fig. S5d and f). Subsequent analysis of two-strain combinations revealed a striking difference in growth responses depending on the interaction between strains. In the analysis of shoot length, the combination of Roc and Pse maintained a level comparable to the untreated group, but the Pse–TFH combination showed significant growth inhibition, recording the shortest shoot length among the analyzed groups (Fig. S5g–j). In contrast, in the analysis of root weight, the Roc–TFH combination recorded a higher value than the untreated group and other treatments, demonstrating that combinations of specific strains can have a positive synergistic effect on root development. When the growth changes according to the composition of multiple strains (≥3 strains) were examined, the growth inhibition phenomenon in certain combinations was more prominent. In particular, the Var–TFH–Pse combination showed the strongest growth inhibition effect, recording significantly lower values in both shoot length (*P* = .002) and shoot weight (*P* = 0) than the untreated group and other multiple strain treatments (Fig. S5k and l). Other multi-strain combinations, including the VTPR treatment, which mixed all four strains, demonstrated stable growth performance, maintaining growth levels statistically similar to the untreated group. Collectively, these results demonstrate that strain combinations within a DMC have a critical impact on early seedling establishment and growth, and that careful consideration of functional complementarity and compatibility among strains is essential for establishing optimal functional DMCs.

### Defined microbial community mediated mitigation of abiotic stress in soybean

The effects of different DMC compositions on soybean salinity stress responses were evaluated using single-strain, two-strain, and multi-strain (≥3 strains) seed coating treatments. Salinity induced damage was quantified using a symptom-based scoring system, from which the salinity injury (SI) index was calculated (Fig. S6a–c). Salt induced damage was quantified using a symptom-based scoring system ranging from 0 to 4, and the SI was calculated according to established criteria (Fig. S6a–c). Analysis of SI values revealed pronounced differences among DMC treatments. In single-strain treatments, Roc exhibited the highest SI value (SI = 84.7), indicating strong sensitivity to salinity stress, whereas TFH showed the lowest SI value (SI = 20.83), reflecting substantial stress mitigation (Fig. S6a). Among two-strain combinations, the Var–Roc treatment resulted in the lowest SI value (SI = 30.53), although the TFH–Pse combination exhibited the highest salt injury (SI = 77.76) (Fig. S6b). In multi-strain treatments (≥3 strains), the Var–Pse–Roc combination and the all-strain DMC showed the lowest SI values (SI = 25.0), whereas the Var–TFH–Pse mixture displayed the highest SI value (SI = 68.03), indicating heightened sensitivity to salinity stress (Fig. S6c). Statistical analyses confirmed significant differences among treatments within each group (single-strain, *P* = .0003677; two-strain, *P* = .01695; multi-strain, *P* = .0009908; Kruskal–Wallis test followed by Conover’s *post hoc* test). TFH single-strain treatment significantly reduced salinity injury compared with the control, whereas Roc single-strain treatment resulted in significantly increased stress sensitivity. Two-strain combinations did not differ significantly from the control, although most multi-strain treatments except the Var–TFH–Pse combination showed significantly attenuated salinity stress relative to the control (Fig. S6a–c).

To further assess physiological responses to salinity stress, chlorophyll fluorescence parameters were measured using leaves immediately below the uppermost fully expanded leaves. Chlorophyll fluorescence measurements revealed that photosynthetic responses to salinity stress depended strongly on DMC composition. Electron transport flux per reaction center (ETo/RC) and dissipated energy flux per reaction center (DIo/RC) were quantified to provide indicators of photosynthetic efficiency and stress-related energy dissipation, respectively (Fig. S6d–i). In single-strain treatments, TFH (ETo/RC = 0.5246) and Pse (ETo/RC = 0.5986) exhibited ETo/RC values comparable to the control (ETo/RC = 0.6402), whereas Var (ETo/RC = 0.3920) and Roc (ETo/RC = 0.4616) showed significantly reduced values (Fig. S6d). Among two-strain combinations, Var–Pse (ETo/RC = 0.7468) and TFH–Roc (ETo/RC = 0.6938) displayed the highest ETo/RC values, indicating enhanced tolerance to salinity stress (Fig. S6f). In contrast, among multi-strain treatments, the Var–TFH–Pse combination exhibited the lowest ETo/RC value (0.3652), whereas the TFH–Pse–Roc mixture showed the highest value (ETo/RC = 0.6746) (Fig. S6h). No significant differences in DIo/RC were detected between single- or two-strain treatments and the control (DIo/RC = 0.9910) (Fig. S6e and g). However, clear divergence emerged among multi-strain treatments, with the Var–TFH–Pse combination showing a marked increase in DIo/RC (2.2654), consistent with elevated energy dissipation under stress conditions (Fig. S6h and i). This treatment also exhibited the lowest ETo/RC among multi-strain groups, indicating reduced photosynthetic electron transport efficiency.

### Differential biocontrol efficacy of single and mixed defined microbial community treatments against *Phytophthora sojae*

**Figure 5:**
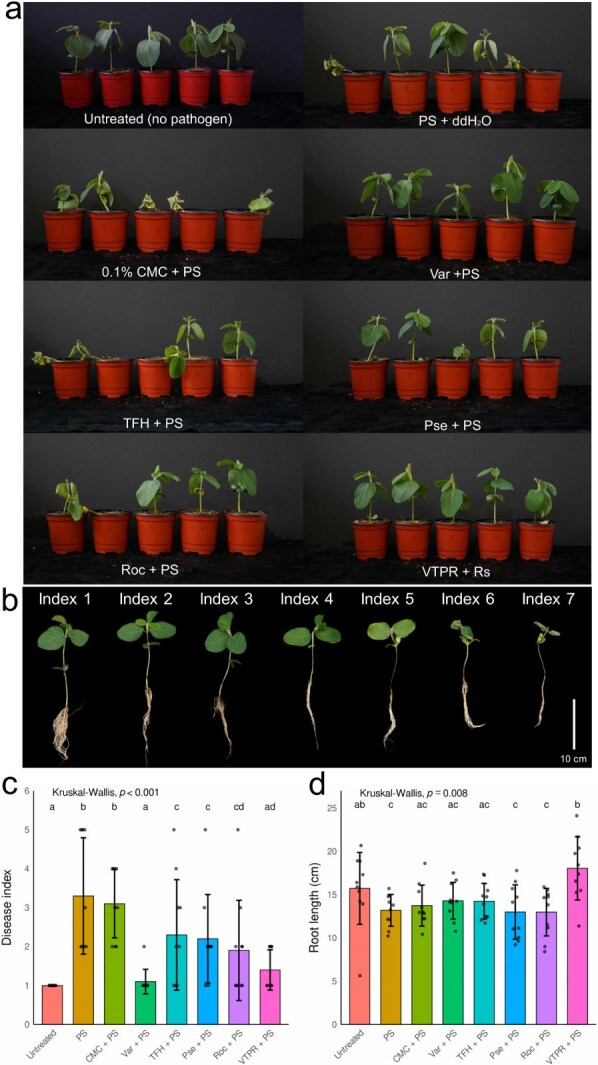
Evaluation of soybean responses to *Phytophthora sojae* infection after the application of synthetic community. DMC bacterial treatments were introduced to soybean plants grown in pots through application of 15 ml bacterial inoculum per plant in days 0, 2, and 4. (a) Plants were infected with *P. sojae* by inflicting lesions on stems using a syringe at Day 6, and subsequently inoculated with 100 μL of mycelial suspension obtained from grinding 7-day old, cultured *P. sojae* mycelia. (b) Disease indices were evaluated 5 days post-inoculation. Disease symptoms were categorized into seven levels as follows: Level 1, no symptoms (healthy plants); Level 2, lesion length < 1 cm; Level 3, lesion length ≥ 1 cm; Level 4, stems wilted and leaves yellowed, but no lodging observed; Level 5, leaves yellowed with lodging; Level 6, leaves completely dried with lodging; Level 7, plants completely dead. Root length measurements was recorded from the initial emergence point of the root to the root tip. Ten replications were performed for each treatment. Statistical analysis of the disease index among treatments. (c–d) Statistical comparisons of root lengths between treatments. PS: *P. sojae*, Var: *V. paradoxus*, TFH: *S. globisporus*, Pse: *P. autotrophica*, roc: *Rhodococcus*. Data were tested for normality and homogeneity of variances using Shapiro–Wilk and Bartlett tests, respectively. Due to violations of these assumptions, statistical evaluations were performed using the non-parametric Kruskal–Wallis test, followed by Conover’s *post hoc* test. Significant differences between treatments are indicated by significance letters.

The biocontrol efficacy of DMC treatments against *Phytophthora sojae*, a soybean root rot pathogen, was evaluated using a controlled biotic stress assay. Treatments included single-strain inoculations and a multi-strain DMC comprising all four strains (Fig. 5a). Control plants were inoculated with the pathogen following either sterile distilled water treatment (DW control) or treatment with 0.1% CMC to account for potential carrier effects.

Disease severity was quantified using a modified disease index (DI) adapted from a previously established scoring system for *P. sojae* infection [17], with symptoms classified into seven disease severity levels (Fig. 5b). Because *P. sojae* infection strongly impairs root and stem development, root length was additionally measured as an indicator of plant growth performance under pathogen pressure (Fig. 5c and d). Comparison of DI values revealed significant differences among treatments (Kruskal–Wallis test, *P* = 6.41 × 10^−7^). Among pathogen-inoculated treatments, Var single-strain treatment exhibited the lowest DI value (DI = 1.1), followed by the all-strain DMC treatment (DI = 1.6), indicating strong suppression of disease symptoms (Fig. 5c). Other single-strain treatments, including TFH; DI = 3.3, Pse; DI = 2.7, and Roc; DI = 2.6, also showed significantly reduced disease severity compared with pathogen-infected controls (DW, DI = 4.9; CMC, DI = 4.7) (Fig. 5c). Root length differed significantly among treatments (*P* = .0083). The all-strain DMC treatment resulted in the greatest mean root length (18.04 cm), exceeding that of the untreated pathogen control (15.72 cm), indicating enhanced growth under pathogen pressure (Fig. 5d). In contrast, the Var single-strain treatment, despite its strong disease suppression, exhibited a shorter average root length (14.29 cm) relative to the all-strain DMC treatment. Conover’s *post hoc* test showed no significant differences in root length between the untreated control and the CMC, Var, or TFH treatments, whereas the all-strain DMC treatment formed a distinct group with significantly increased root length (Fig. 5d).

## Discussion

The flower stage represents a critical developmental window in soybean, as it determines reproductive success and seed formation [4, 5]. Floral tissues are physiologically active and characterized by elevated nutrient availability, although remaining continuously exposed to external microbial sources such as air, insects, and surrounding plant surfaces. This ecological context likely explains the high variability and elevated diversity observed in flower-associated communities. Similar patterns of unstable and heterogeneous floral microbiota have been reported in other plant species, where frequent immigration limits strong host-mediated selection [9, 10].

The transition from vegetative to reproductive growth in soybean represents a pivotal ecological shift that dictates the composition of the seed-associated microbiota. In this study, we examined structural changes in microbial communities during soybean reproductive development, with a particular focus on the floral and pod stages. Overall microbial diversity declined progressively during development advanced, with a pronounced reduction beginning at R2, followed by dominance of a limited number of taxa during R3–R5 stages. Such patterns suggest that microbial community assembly during reproduction is increasingly shaped by host associated filtering and competitive interactions rather than random colonization, consistent with ecological filtering processes described in other plant-associated microbiota [8]. With development progressed into early pod formation, microbial community structure shifted sharply. Principal coordinate analysis revealed clear separation of R3 communities from earlier floral stages, accompanied by reduced within-stage variability and enrichment of specific taxa. Pod enclosure likely restricts microbial influx and alters physicochemical conditions within the tissue, favoring microorganisms adapted to more isolated internal environments. Comparable reductions in microbial complexity under enclosed or restricted conditions have been observed in other endophytic systems, where physical isolation promotes stable colonization by a narrow subset of microbes [12, 39].

Taxaturn model using βNTI and βRC metrics provided further insight into the processes shaping these compositional shifts. During the floral stage, βNTI values remained close to neutral expectations, indicating limited phylogenetic structuring and a strong influence of stochastic processes including dispersal and drift. These patterns are consistent with the open nature of flowers and their continuous exposure to diverse microbial sources [7, 40]. This “open-gate” strategy may be essential for capturing a diverse suite of microbes, some of which are later refined for vertical transmission. In contrast, microbial communities associated with pod stages exhibited signatures of dispersal limitation, with βRC values exceeding 0.95 despite |βNTI| values below 2. Restricted microbial movement following pod enclosure likely leads to independent community development and divergence from earlier stages, even in the absence of strong environmental gradients [41]. The physical enclosure of the pod creates an isolated “island” environment, restricting further microbial influx and forcing internal competition. This isolation promotes the stable colonization of a specialized subset of endophytes that are physiologically adapted to the unique physicochemical conditions of the developing seeds. Stage resolved analyses further revealed that bud stage samples harbored distinct microbial assemblages compared with open flowers and later pod stages. Bud tissues represent early reproductive organs with concentrated nutrient pools and limited exposure to the external environment. Similar stage specific microbial signatures have been observed in other crops, where early reproductive tissues support lower diversity but more specialized microbiomes driven by host metabolic profiles [42, 43]. These observations highlight that microbial community dynamics during reproduction are closely linked to host physiology. Interactions including competition, antibiosis, and differential nutrient utilization likely intensify during tissues mature, contributing to repeated restructuring of microbial communities across developmental stages [44]. The consistent enrichment of Pseudonocardiaceae during the R3–R5 supports this interpretation and suggests that their enrichment reflects selective advantages under pod associated conditions rather than stochastic effects. Members of the family Pseudonocardiaceae are widely documented endophytic actinomycetes in diverse plant hosts and are frequently associated with antimicrobial activity and stress tolerance [45]. The dominance of taxa represented by *Pseudonocardia carboxydivorans*, which can suppress competitors through antibiosis [46], suggests that the pod environment selects for a “guardian” microbiota. Their consistent presence during soybean pod development suggests a stable association that may contribute to host protection during seed formation. This selective enrichment likely serves a dual purpose: protecting the developing seeds from opportunistic pathogens and ensuring that beneficial functional traits are prioritized for transmission to the next generation.

The functional characterization of the identified keystone taxa reveals a sophisticated system of niche partitioning and functional complementarity, where the division of labor combining *Variovorax*, *Streptomyces*, and *Pseudonocardia,* optimizes the collective metabolic output for the host [32]. *Variovorax* has been shown to modulate plant growth in complex microbiota contexts, highlighting its ecological relevance to host phenotype [47]. *Streptomyces* species, including *S. globisporus*, have been associated with suppression of soil-borne pathogens and growth-promotion effects [48], and *Pseudonocardia* has been studied for antifungal potential and antibiotic production [49]. Our observation that specific mixtures like Var–TFH–Pse inhibited seedling growth suggests that mismatched microbial interactions can trigger antagonistic signaling or impose an energetic burden on the host during the sensitive germination window [16]. This balance of microbial “social” behavior becomes particularly critical under environmental pressure, where multi-strain DMCs provide a superior level of ecological buffering compared to single-strain inoculants. Even taxa that appear sensitive in isolation contribute to a collective resilience within the full consortium, stabilizing photosynthetic efficiency and minimizing stress-induced energy dissipation under salinity [15]. The efficacy of the all-strain DMC against *P. sojae* demonstrates that holistic plant fitness requires synergizing direct pathogen antagonism with compensatory growth. Although single-taxa treatments like *Variovorax* excelled at suppressing disease symptoms, only the multi-member consortium successfully restored root development under pathogen pressure. This reinforces the model that a robust microbiota-based strategy must integrate both defensive and growth-promoting functions to ensure functional persistence and host productivity across diverse biological and environmental stressors.

A central question in plant microbial ecology is how maternal microbes successfully navigate the transition into the seed. We propose that the soybean endocarp serves a critical anatomical conduit for this generational transmission. The endocarp maintains an intimate, lifelong physical connection with the seed locule [13, 50]. Anatomical studies have shown that the soybean endocarp comprises tightly packed cells that remain in close physical contact with developing seeds maturation [50]. This intimate spatial association suggests that the endocarp may function constitute a physical interface facilitating microbial movement from maternal tissues into the seed environment. The observed “reappearance” of floral-associated taxa in the late pod stages, following an initial decline, suggests a model of internal redistribution rather than de novo colonization. In this model, the endocarp functions a reservoir that protects a conserved subset of the floral microbiota, allowing it to bypass external environmental filters and colonize the maturing seed [9]. This structural-ecological coupling ensures that the most fit microbial lineages are vertically transmitted to the next generation [51].

Taken together, our findings indicate that the R2–R5 developmental window is the most critical period for the assembly of the soybean seed microbiota. The shift from a stochastic floral pool to a deterministic, dispersal limited pod community provides a clear blueprint for microbiota-based interventions. Future efforts to enhance crop performance through inoculants should target this specific “transmission window,” utilizing strains with the metabolic and competitive traits (e.g. stress resistance, antibiosis) necessary to survive the host’s rigorous ecological filters [15, 16].

## Supplementary Material

Supplementary_material_wrag183

## Data Availability

Pyrosequencing data for flower samples have been deposited in the NCBI Bioproject accession number PRJNA1107009 [https://www.ncbi.nlm.nih.gov/bioproject/PRJNA1107009/]. The source data underlying this study are provided as Supplementary Data files (Supplementary dataset 1 and dataset 2). All other relevant data and resources are available from the authors.

## Code availability

Analyses of microbial community composition were carried out with DADA2 platform (version 1.20.0) as https://benjjneb.github.io/dada2/ReleaseNotes_1_20.html. The source code of R for data analyses is available on https://github.com/ekfks0125-wq/Soybean_small_large_scale/tree/main.
