# Supplementary material for: Deterministic recruitment limitation during soybean pod development shapes seed-transmitted microbiota assembly
Source: ISME J. 2026 Jul 15;20(1):wrag183. doi: 10.1093/ismejo/wrag183 (PMC13477215; doi:10.1093/ismejo/wrag183)
Supplement: Supplementary file 1 [file supplementary_material_wrag183.zip › Final Supplementary Information.docx]

**Supplementary Information**

*for*

**Deterministic Recruitment Limitation during Soybean Pod Development Shapes Seed-Transmitted Microbiota Assembly**

*Kim et al.*

This file includes 10 tables and 6 figures.

| Type | Stage | Sample | Biosample |
| --- | --- | --- | --- |
| Small-scale  (*n* = 5) | Flower stage | FB (Flower bud) | SAMN41158276  (01-FunBio-FTPs-21Mar) |
|  |  | BF (Beginning flower) |  |
|  |  | DF (Developing flower) |  |
|  |  | OF (Opening flower) |  |
|  | Pod stage | R2 (Pollinated flower/initiation pot) |  |
|  |  | R3 (Beginning pod) |  |
|  |  | R4 (Developing seed) |  |
|  |  | R5 (Mature seed) |  |
|  | Plant | Young stem (V X) |  |
|  |  | Old stem (R X) |  |
| Large-scale  (*n* = 30) | Flower stage | Bud (Flower bud) | SAMN41158283  (02-FunBio-FTPs-21Mar) |
|  |  | Flower (Opening flower) |  |
|  | Pod stage | R3 (Beginning pod) |  |
|  |  | R5 (Mature seed) |  |

**Supplementary Table 1.** Sampling stage design for small-scale and large-scale microbiota analyses

**Supplementary Table 2**. Concentrations and dispensing volumes for DMC preparation

| Group | Treatments | Split (µL/ 10 g) | Per-strain cell density (CFU/mL) |
| --- | --- | --- | --- |
| Solo | Var | 100 | 1×10⁷ |
|  | Roc | 100 | 1×10⁷ |
|  | TFH | 100 | 1×10⁶ |
|  | Pse | 100 | 1×10⁶ |
| Double | Var + Roc | 50 + 50 | 1×10⁷ / 1×10⁷ |
|  | Var + TFH | 50 + 50 | 1×10⁷ / 1×10⁶ |
|  | Var + Pse | 50 + 50 | 1×10⁷ / 1×10⁶ |
|  | TFH + Roc | 50 + 50 | 1×10⁶ / 1×10⁷ |
|  | Pse + Roc | 50 + 50 | 1×10⁶ / 1×10⁷ |
|  | TFH + Pse | 50 + 50 | 1×10⁶ / 1×10⁶ |
| Three or more | Var + TFH + Pse | ≈33.33 ×3 | 1×10⁷ / 1×10⁶ / 1×10⁶ |
|  | Var + TFH + Roc | ≈33.33 ×3 | 1×10⁷ / 1×10⁶ / 1×10⁷ |
|  | Var + Pse + Roc | ≈33.33 ×3 | 1×10⁷ / 1×10⁶ / 1×10⁷ |
|  | TFH + Pse + Roc | ≈33.33 ×3 | 1×10⁶ / 1×10⁶ / 1×10⁷ |
|  | Var + TFH + Pse + Roc | 25 ×4 | 1×10⁷ / 1×10⁶ / 1×10⁶ / 1×10⁷ |
| Control | Untreated | — | — |

**Supplementary Table 3.** DMC treatments and strain suspension scheme for biotic stress experiments

| Treatments | DMC suspension OD_600_ (pre-dilution) * | Application rate (per plant) | *Phytophthora sojae* |
| --- | --- | --- | --- |
| Var (single) | 0.5 | 15 mL × 3 | Yes |
| Pse (single) | 0.5 | 15 mL × 3 | Yes |
| TFH (single) | 0.3 | 15 mL × 3 | Yes |
| Roc (single) | 0.3 | 15 mL × 3 | Yes |
| ALL (multi) | Var 0.5; Pse 0.5; TFH 0.3; Roc 0.3 † | 15 mL × 3 | Yes |
| CMC control | — | 15 mL × 3 | Yes |
| DW control | — | 15 mL × 3 | Yes |
| Pathogen-free control | — | 15 mL × 3 | No |

* OD values listed are prior to the 1:10 dilution in 0.1% CMC used to prepare working suspensions.

† Equal-volume mixing indicates volumetric mixing of the four strain suspensions at the indicated pre-dilution ODs in a 1:1:1:1 ratio (v/v).

**Supplementary Table 4.** amplicon sequence variants (ASVs) number identifiers to candidate keystone taxa prioritized from the soybean microbial community dataset

| Taxa | ASVs number |
| --- | --- |
| *Pseudonocardia* | ASV_162 |
| *Rhodanobacter* | ASV_9 |
| *Rhodococcus* | ASV_3 |
|  | ASV_281 |
|  | ASV_643 |
| *Streptomyces* | ASV_11 |
|  | ASV_398 |
| *Variovorax* | ASV _43 |
|  | ASV_117 |
|  | ASV_135 |
|  | ASV_151 |
|  | ASV_153 |
|  | ASV_160 |
|  | ASV_168 |
|  | ASV_169 |
|  | ASV_174 |
|  | ASV_178 |

**Supplementary Table 5.** Taxonomic and stage occurrence of keystone taxa

| Class or order | Order or family | Genus | Stage |
| --- | --- | --- | --- |
| Actinobacteria | Nocardiaceae | *Rhodococcus* | Bud, R3 |
| Actinobacteria | Pseudonocardiaceae | *Pseudonocardia* | Bud, FL |
| Actinobacteria | Streptomycetaceae | *Streptomyces* | Bud - R5 |
| Burkholderiales | Comamonadaceae | *Variovorax* | R3 |
| Xanthomonadales | Rhodanobacteraceae | *Rhodanobacter* | Bud, FL |

**Supplementary Table 6.** Network centrality (betweenness) of taxa in the inferred interaction network

| Name | Betweenness |
| --- | --- |
| Comamonadaceae | 3503 |
| *Cutibacterium* | 2409 |
| *Rhodococcus* | 1517 |
| *Acidisoma* | 1288 |
| Isosphaeraceae | 1117 |
| *Ralstonia* | 797 |
| Rhodanobacteraceae | 747 |
| *Mycobacterium* | 741 |
| Vermiphilaceae | 481 |
| *Rhodanobacter* | 473 |
| *Pseudonocardia* | 326 |
| *Streptomyces* | 280 |
| *Leptolyngbya* PCC-6306 | 218 |
| *Streptococcus* | 182 |
| *Scytonema* UTEX 2349 | 161 |

**Supplementary Table 7.** Network centrality (PageRank) of taxa in the inferred interaction network

| Name | Pagerank |
| --- | --- |
| *Rhodococcus* | 0.035 |
| *Chroococcidiopsis* SAG 2023 | 0.024 |
| *Scytonema* UTEX 2349 | 0.015 |
| Burkholderiaceae | 0.014 |
| Comamonadaceae | 0.012 |
| Vermiphilaceae | 0.009 |
| *Leptolyngbya* PCC-6306 | 0.009 |
| *Gaiella* | 0.008 |
| Isosphaeraceae | 0.008 |
| *Taibaiella* | 0.008 |
| *Blastococcus* | 0.007 |
| *Kaistia* | 0.006 |
| *Methylobacterium-Methylorubrum* | 0.006 |
| Neisseriaceae | 0.006 |
| HSB OF53-F07 | 0.006 |

**Supplementary Table 8**. Differentially abundant taxa between stages (LDA-based ranking)

| ASVs | RelRareAbundance | | Organism | | Family | | Genus | | LDAmean | | *P-*value | | FDR | |
| --- | --- | --- | --- | --- | --- | --- | --- | --- | --- | --- | --- | --- | --- | --- |
| ASV_2 | 44.4206 | Flower | | f__Comamonadaceae | | - | | 5.01 | | 1.85E-05 | | 0.000137 | |  |
| ASV_7 | 3.969957 | Flower | | f__Nocardiaceae | | g__Rhodococcus | | 3.95 | | 6.05E-09 | | 7.14E-08 | |  |
| ASV_22 | 0 | Flower | | f__Comamonadaceae | | - | | 2.55 | | 0.000148 | | 0.000792 | |  |
| ASV_4 | 0.107296 | Pod | | f__Comamonadaceae | | - | | 4.10 | | 1.58E-20 | | 6.99E-19 | |  |
| ASV_5 | 0 | Pod | | f__Rhodanobacteraceae | | g__Rhodanobacter | | 3.99 | | 4.58E-23 | | 5.55E-21 | |  |
| ASV_6 | 0.107296 | Pod | | f__Burkholderiaceae | | g__Ralstonia | | 3.85 | | 6.11E-14 | | 9.84E-13 | |  |
| ASV_8 | 0 | Pod | | f__Rhodanobacteraceae | | g__Rhodanobacter | | 3.75 | | 6.27E-23 | | 5.55E-21 | |  |
| ASV_9 | 0 | Pod | | f__Burkholderiaceae | | g__Burkholderia-Caballeronia-Paraburkholderia | | 3.42 | | 2.06E-07 | | 2.15E-06 | |  |
| ASV_11 | 0 | Pod | | f__Vermiphilaceae | | - | | 3.60 | | 3.94E-05 | | 0.000279 | |  |
| ASV_12 | 0 | Pod | | f__Mycobacteriaceae | | g__Mycobacterium | | 3.41 | | 1.22E-06 | | 1.20E-05 | |  |
| ASV_13 | 0.107296 | Pod | | f__Propionibacteriaceae | | g__Cutibacterium | | 3.45 | | 1.00E-16 | | 2.54E-15 | |  |
| ASV_14 | 0 | Pod | | f__Rhodanobacteraceae | | g__Rhodanobacter | | 3.05 | | 3.19E-10 | | 4.03E-09 | |  |
| ASV_15 | 0 | Pod | | f__Rhodanobacteraceae | | - | | 3.14 | | 3.77E-11 | | 5.13E-10 | |  |
| ASV_16 | 0 | Pod | | f__Rhodanobacteraceae | | g__Rhodanobacter | | 3.04 | | 5.04E-06 | | 4.46E-05 | |  |
| ASV_17 | 0 | Pod | | f__Rhodanobacteraceae | | g__Rhodanobacter | | 3.032371 | | 1.01E-05 | | 7.76E-05 | |  |
| ASV_19 | 0 | Pod | | f__Rhodanobacteraceae | | - | | 2.78 | | 7.65E-05 | | 0.000468 | |  |
| ASV_20 | 0 | Pod | | f__Rhodanobacteraceae | | - | | 2.62 | | 0.000103 | | 0.000567 | |  |
| ASV_21 | 0 | Pod | | f__Rhodanobacteraceae | | g__Rhodanobacter | | 2.77 | | 2.46E-06 | | 2.29E-05 | |  |
| ASV_25 | 0 | Pod | | f__Rhodanobacteraceae | | g__Chujaibacter | | 2.78 | | 0.001911 | | 0.008458 | |  |
| ASV_26 | 0 | Pod | | f__Rhodanobacteraceae | | g__Chujaibacter | | 2.72 | | 0.001017 | | 0.004614 | |  |
| ASV_30 | 0 | Pod | | f__Streptomycetaceae | | - | | 2.50 | | 0.000282 | | 0.001429 | |  |

**Supplementary Table 9.** DMC strains accession numbers and resources used in this study

| Species | Name | Origin | Strain ID | Genome sequencing | References |
| --- | --- | --- | --- | --- | --- |
| *Pseudonocardia autotrophica* | Pse | Soil | NBRC12743 | PacBio | [1] |
| *Rhodanobacter spathiphylli* | Rod | Root | B39^T^ | 454 GS-FLX Titanium; Illumina MiSeq | [2] |
| *Rhodococcus* sp. | Roc | Sweet potato leaf | KB6 | Illumina HiSeq | [3] |
| *Variovorax paradoxus* | Var | Root nodule | NBRC15149 | 454 GS-FLX Titanium; Illumina MiSeq | [4] |
| *Streptomyces globisporus* | TFH | Tomato flower | TFH56 | PacBio | [5] |

1. Yoshida K, Yasutake Y, Tamura T. Complete genome sequence of an efficient vitamin d3-hydroxylating bacterium, *Pseudonocardia autotrophica* NBCR 12743. *Microbiol Resour Announc*. 2018;**7**:10.1128/mra. 01105–18 https://doi.org/https://doi.org/10.1128/mra.01105-18

2. De Clercq D, Van Trappen S, Cleenwerck I *et al.* *Rhodanobacter spathiphylli* sp. nov., a gammaproteobacterium isolated from the roots of *Spathiphyllum* plants grown in a compost-amended potting mix. *Int J syst Evol Microbiol*. 2006;**56**:1755–59 https://doi.org/https://doi.org/10.1099/ijs.0.64131-0

3. Hong CE, Jeong H, Jo SH *et al.* A leaf-inhabiting endophytic bacterium, *Rhodococcus* sp. Kb6, enhances sweet potato resistance to black rot disease caused by *Ceratocystis fimbriata*. *J Microbiol Biotechnol*. 2016;**26**:488–92 https://doi.org/https://doi.org/10.4014/jmb.1511.11039

4. Ville CJN, Orwin PM. Completed genomes from *Variovorax* provide insight into genome diversification through horizontal gene transfer. *Curr Res Microb Sci*. 2025:100497 https://doi.org/https://doi.org/10.1016/j.crmicr.2025.100497

5. Cho G, Kwak Y-S. Evolution of antibiotic synthesis gene clusters in the *Streptomyces globisporus* THF56, isolated from tomato flower. *G3: Genes Genomes Genet*. 2019;**9**:1807–13 https://doi.org/https://doi.org/10.1534/g3.119.400037

**Supplementary Table 10.** Functional trait matrix of DMC strains (qualitative scoring)

| Strains | Chitinase | Protease | Phosphate-solubilizing | Siderophore | IAA production | Nitrogen fixation |
| --- | --- | --- | --- | --- | --- | --- |
| *Pseudonorcadia* (Pse) | - | - | - | + | - | - |
| *Rhodanobacter* (Rod) | - | - | ++ | - | ++ | - |
| *Rhodococcus* (Roc) | - | - | - | - | - | ++ |
| *Streptomyces* (TFH) | + | ++ | - | + | - | - |
| *Variovorax* (Var) | - | - | - | +++ | - | - |

**Supplementary Figure 1. Microbiome diversity, assembly processes across soybean small scale.** Amplicon sequence variants (ASVs) were generated from 16S rRNA gene amplicon sequencing using the DADA2 pipeline (v1.20.0) in R (v4.1.3), including quality filtering, error correction, paired-end read merging, and chimera removal. Taxonomic classification was performed against the SILVA SSU database (v138) using the IDTAXA classifier. (**a**) Rarefaction curves were generated with Amplicon sequence variant (ASV) was calculated at increasing sequencing depths using the phyloseq and vegan packages in R (v4.4.3). Community richness and evenness were assessed with ggiNEXT (v2.0.20). (**b, c**) Alpha diversity indices (ACE, Fisher, InvSimpson, Observed ASVs, Shannon, and Simpson) and Bray–Curtis–based beta diversity was calculated using the vegan (v2.6-2) and phyloseq (v1.36.0) packages. Statistical significance was evaluated using Kruskal–Wallis tests (*P* < 0.05).

**Supplementary Figure 2. Small-scale community variation patterns across soybean stages.** Microbial community dissimilarities at a small spatial scale were quantified using Bray–Curtis distance matrices calculated from ASV-based relative abundance tables. (**a**) Principal Coordinates Analysis (PCoA) was applied to visualize differences in microbial community composition among flower, pod, and stem tissues across developmental stages. (**b**) Linear regression models were fitted to examine associations between microbial community variation and developmental stage, and model comparisons were performed using analysis of variance (ANOVA) to evaluate differences among groups. (**c**-**h**) Ternary plots were constructed to display the relative distribution of microbial taxa among three selected sample groups, with marker sizes representing mean relative abundance and colors assigned according to phylum-level classification. Taxa with low relative abundance (<0.1%) were excluded or combined into others.

**Supplementary Figure 3. Microbiome diversity and community patterns across soybean at a large scale.** Amplicon sequence variants (ASVs) were obtained from 16S rRNA gene amplicon sequencing data processed using the DADA2 pipeline (v1.20.0) in R, including quality filtering, denoising, merging of paired-end reads, and removal of chimeric sequences. Taxonomic assignment was performed using the SILVA SSU reference database (v138) with the IDTAXA. (**a**) Alpha diversity indices, including ACE, Fisher, InvSimpson, Observed ASVs, Shannon, and Simpson, were calculated for each sample, and beta diversity was estimated based on Bray–Curtis dissimilarity using the vegan (v2.6-2) and phyloseq (v1.36.0) packages. Differences in diversity metrics among sample groups were evaluated using Kruskal–Wallis tests, with statistical significance defined as *P* < 0.05. (**b**) Beta diversity was assessed using Bray–Curtis dissimilarity to compare microbial community composition among samples. Visualize differences in community structure across soybean developmental stages. Group-level differences in community composition were evaluated based on Bray–Curtis distances. Dissimilarity matrices were calculated from ASV-based relative abundance tables using the vegan and phyloseq packages in R (v 4.4.3).

**Supplementary Figure 4. Conceptual illustration of inferred microbial source–sink relationships during soybean seed development.** (**a**) SourceTracker analysis across soybean reproductive stages. Source tracking was conducted using the SourceTracker package, following the implementation described in the original repository (https://github.com/danknights/sourcetracker/). ASV-based taxonomic tables were generated, in which flower bud samples were defined as source communities, flower tissues and pod samples collected at the R3 and R5 stages were defined as sink communities. (**b, c**) All graphical elements were created and assembled using Affinity Designer (version 1.10), based on quantitative results obtained from microbial community analyses.

**Supplementary Figure 5. Effects of DMC treatments on soybean early growth.** (**a-b**) Experimental schematic illustrating the workflow of DMC-coated seed assays. Soybean seeds (cv. Daepung) were surface-sterilized, coated with individual or combined DMC strains using a 0.1% carboxymethyl cellulose (CMC) carrier, and germinated on sterile plates under dark conditions for 3 days. Germinated seedlings were subsequently transferred to soil and grown for 10 days prior to phenotypic assessment. Sampling time points for germination, growth measurement, and endophytic colonization are indicated. (**c-f**) Effects of single-strain DMC treatments on early seedling performance under soil conditions at 10 days post-germination (*n* = 7). Shoot length (**c**), shoot fresh weight (**d**), root length (**e**), and root fresh weight (**f**) were quantified. Treatments included individual inoculation with *Variovorax paradoxus* NBRC15149 (Var), *Streptomyces globisporus* TFH56 (TFH), *Pseudonocardia autotrophica* DMS535 (Pse), and *Rhodococcus* sp. KCB6 (Roc), as well as an untreated control. (**g–j**) Effects of two-strain DMC combinations on seedling growth parameters at 10 days post-germination (*n* = 7). Shoot length (**g**), shoot fresh weight (**h**), root length (**i)**, and root fresh weight (**j**) were measured following seed coating with all possible pairwise combinations of DMC strains. (**k–l**) Effects of multi-strain DMC combinations (three or more strains) on soybean seedling growth traits (*n* = 7). Growth parameters were quantified as described above to evaluate potential synergistic or antagonistic interactions among DMC members during early plant development. All data are presented as boxplots showing median values, interquartile ranges, and individual biological replicates. Statistical significance among treatments was assessed using Kruskal–Wallis tests followed by Conover’s post hoc comparisons. Different letters indicate significant differences between treatment groups at *P* < 0.05.

**Supplementary Figure 6. Salinity stress response of soybean after DMC seed coating assessed by injury index and chlorophyll fluorescence analysis.** Seeds were vernalized on plates for 3 days after coating with specific DMC strains and plants were transferred to pots at 7-days post germination. Salt stress treatment was conducted via application of 160 mM NaCl. SI (Salinity Index) measurements were recorded at 10-days post treatment following the onset of control plant senescence and dedication. (**a**) Solo treatments, (**b**) Double treatments, and (**c**) Three or more combined treatments. The plants were grouped in replicates of three and the formula SI = (N1 + N2 x 2 + N3 x 3 + N4 x 4)/(N x 4) x 100 were used for measuring SI values. Var: *V. paradoxus* NBRC15149, TFH: *S. globisporus* TFH56, Pse: *P. autotrophica* DMS535, Roc: *Rodocuccus* sp. KCB6. (**d-f**) Statistical comparisons of the ETo/RC among different treatment groups. (**g-i**) Statistical differences of the DIo/RC among various treatment groups. Graphs depict comparisons between control and solo treatments (**d** and **e**), control versus double-combination treatments (**f** and **g**), and control versus combinations of three or more treatments (**h** and **i**). Var: *V. paradoxus* NBRC15149, TFH: *S. globisporus* TFH56, Pse: *P. autotrophica* DMS535, Roc: *Rodococcus* sp. KCB6. Data were evaluated for normality and homogeneity of variances using the Shapiro-Wilk and Bartlett tests, respectively. Due to the violation of these assumptions, statistical analyzes were conducted using the non-parametric Kruskal–Wallis test, followed by Conover’s post-hoc test. Significant differences between treatments are indicated by significance letters.
